# Platelet-activating factor antagonists enhance intracellular degradation of amyloid-β_42_ in neurons via regulation of cholesterol ester hydrolases

**DOI:** 10.1186/alzrt245

**Published:** 2014-03-13

**Authors:** Charlotte Simmons, Victoria Ingham, Alun Williams, Clive Bate

**Affiliations:** 1 Department of Pathology and Pathogen Biology, Royal Veterinary College, Hawkshead Lane, North Mymms, Herts AL9 7TA, UK; 2 Department of Veterinary Medicine, University of Cambridge, Madingley Road, Cambridge CB3 OES, UK

## Abstract

**Introduction:**

The progressive dementia that is characteristic of Alzheimer’s disease is associated with the accumulation of amyloid-beta (Aβ) peptides in extracellular plaques and within neurons. Aβ peptides are targeted to cholesterol-rich membrane micro-domains called lipid rafts. Observations that many raft proteins undertake recycling pathways that avoid the lysosomes suggest that the accumulation of Aβ in neurons may be related to Aβ targeting lipid rafts. Here we tested the hypothesis that the degradation of Aβ by neurons could be increased by drugs affecting raft formation.

**Methods:**

Primary neurons were incubated with soluble Aβ preparations. The amounts of Aβ_42_ in neurons or specific cellular compartments were measured by enzyme-linked immunosorbent assay. The effects of drugs on the degradation of Aβ_42_ were studied.

**Results:**

Aβ_42_ was targeted to detergent-resistant, low-density membranes (lipid rafts), trafficked via a pathway that avoided the lysosomes, and was slowly degraded by neurons (half-life was greater than 5 days). The metabolism of Aβ_42_ was sensitive to pharmacological manipulation. In neurons treated with the cholesterol synthesis inhibitor squalestatin, less Aβ_42_ was found within rafts, greater amounts of Aβ_42_ were found in lysosomes, and the half-life of Aβ_42_ was reduced to less than 24 hours. Treatment with phospholipase A_2_ inhibitors or platelet-activating factor (PAF) antagonists had the same effects on Aβ_42_ metabolism in neurons as squalestatin. PAF receptors were concentrated in the endoplasmic reticulum (ER) along with enzymes that constitute the cholesterol ester cycle. The addition of PAF to ER membranes triggered activation of cholesterol ester hydrolases and the release of cholesterol from stores of cholesterol esters. An inhibitor of cholesterol ester hydrolases (diethylumbelliferyl phosphate) also increased the degradation of Aβ_42_ in neurons.

**Conclusions:**

We conclude that the targeting of Aβ_42_ to rafts in normal cells is a factor that affects its degradation. Critically, pharmacological manipulation of neurons can significantly increase Aβ_42_ degradation. These results are consistent with the hypothesis that the Aβ-induced production of PAF controls a cholesterol-sensitive pathway that affects the cellular localization and hence the fate of Aβ_42_ in neurons.

## Introduction

The amyloid hypothesis of Alzheimer’s disease (AD) pathogenesis maintains that the primary event is the production of specific C-terminal amyloid-beta (Aβ) peptides following the abnormal proteolytic cleavage of the amyloid precursor protein [1]. Aβ oligomers demonstrate disease-specific accumulation in human brain and cerebrospinal fluid [2]. The accumulation of Aβ peptides leads to the subsequent disruption of neuronal processes, abnormal phosphorylation of tau [3], and synapse degeneration [4]. Currently, soluble Aβ_42_ oligomers are regarded as potent neurotoxins [5,6].

Neurodegeneration is preceded by the intraneuronal accumulation of Aβ [7,8]. The chronic nature of AD suggests that it is a slow accumulation of Aβ that triggers neurodegeneration and hence the clinical symptoms. Whereas the factors that affect the production of the Aβ have been studied extensively, the capacity of neurons to degrade Aβ once it has been formed has received less attention. Thus, the accumulation of Aβ within neurons may result from a slow rate of degradation. Aβ peptides can be degraded by proteases, including neprilysin [9], insulysin [10], cathepsin B [11], and acyl peptide hydrolase [12]. The observation that Aβ was degraded more quickly in microglial cells than in neurons (unpublished data) raised the question of whether the rate of degradation of Aβ within neurons could be increased.

Studies that use synthetic Aβ preparations may be compromised by their propensity to self-aggregate into a wide variety of oligomer sizes and conformations. The polymorphic nature of Aβ aggregates suggests that there exist disease-relevant conformations of Aβ but that other conformations are less toxic [13,14]. It is difficult to control the size and conformation of synthetic Aβ_42_ oligomers in aqueous medium and consequently it is not clear which of the Aβ conformations are responsible for specific biological properties. To overcome this problem, conditioned media from 7PA2 cells (7PA2-CM) which contain naturally secreted Aβ oligomers [15] were used in this study. The Aβ oligomers secreted by these cells are sodium dodecyl sulphate (SDS)-stable, as are the Aβ oligomers found within the cerebrospinal fluid of patients with AD [16-18]. Although 7PA2-CM includes other amyloid precursor protein (APP) metabolites, including Aβ_40_ and p3, we decided to specifically measure Aβ_42_ peptides because of the close association of this isoform with disease.

Aβ_42_ peptides are found in detergent-resistant, cholesterol-dense membrane micro-domains which are commonly referred to as rafts [19-21]. Since many raft-associated proteins traffic through cells via recycling pathways that avoid the lysosomes [22,23], we hypothesized that the targeting of Aβ_42_ to rafts caused it to avoid the lysosomes and degradation. Thus, the targeting of Aβ_42_ to rafts may contribute to their gradual accumulation within neurons and subsequently their toxic effects. In this study, non-toxic concentrations of Aβ_42_ were taken up by cultured neurons and targeted to detergent-resistant membranes (DRMs) (lipid rafts). As a consequence, it was slowly degraded and had a half-life of greater than 5 days. As the formation and function of lipid rafts is dependent upon the amount of cholesterol within cell membranes [24-26], the effects of the cholesterol synthesis inhibitor squalestatin [27] on the cellular targeting of Aβ_42_ were studied. Here, we show that, in neurons treated with squalestatin, significantly less Aβ_42_ was targeted to rafts and more was found within lysosomes. Consequently, in squalestatin-treated neurons, the half-life of Aβ_42_ was reduced to less than 24 hours. Such results indicate that the pharmacological manipulation of neurons can alter the clearance of Aβ_42_.

## Methods

### Primary neuronal cultures

Cortical neurons were prepared from the brains of mouse embryos (day 15.5). Neurons were plated at 10^6^ cells per well in six-well plates in Ham’s F12 containing 5% fetal calf serum for 2 hours. Cultures were shaken (600 rpm for 5 minutes), and non-adherent cells were removed by two washes in phosphate-buffered saline (PBS). Neurons were subsequently grown in neurobasal medium containing B27 components supplemented with 5 nM brain-derived nerve growth factor for 10 days. Immunohistochemistry showed that the cells were greater than 95% neurofilament-positive. Less than 3% stained for glial fibrillary acidic protein (astrocytes) or for F4/80 (microglial cells). Neurons were pre-treated with test compounds for 3 hours before the addition of Aβ preparations, and the amount of Aβ_42_ in treated neurons was measured at specific time points thereafter. All experiments were performed in accordance with European regulations (European community Council Directive, 1986, 56/609/EEC) and approved by the Royal Veterinary College ethics committee.

### Cell extracts

Neurons were washed twice with PBS and homogenized in a buffer containing 10 mM Tris-HCl, pH 7.4, 150 mM NaCl, 10 mM EDTA, 0.5% Nonidet P-40, 0.5% sodium deoxycholate, 0.2% SDS, and mixed protease inhibitors (4-(2-Aminoethyl) benzenesulfonyl fluoride hydrochloride (AEBSF), Aprotinin, Leupeptin, Bestatin, Pepstatin A, and E-46) (Sigma-Aldrich, St. Louis, MO, USA) at 10^6^ cells/mL. Nuclei and large fragments were removed by centrifugation (300 *g* for 5 minutes).

### Isolation of detergent-resistant membranes

These membranes were isolated by their insolubility in non-ionic detergents as described [28]. Briefly, cells were homogenized in an ice-cold buffer containing 1% Triton X-100, 10 mM Tris-HCl, pH 7.2, 150 mM NaCl, 10 mM EDTA, and mixed protease inhibitors at 10^6^ cells/mL, and nuclei and large fragments were removed by centrifugation (300 *g* for 5 minutes at 4°C). The post-nuclear supernatant was incubated on ice (4°C) for 1 hour and centrifuged (16,000 *g* for 30 minutes at 4°C). The supernatant was reserved as the detergent-soluble membrane; the insoluble pellet was homogenized in an extraction buffer containing 10 mM Tris-HCl, pH 7.4, 150 mM NaCl, 10 mM EDTA, 0.5% Nonidet P-40, 0.5% sodium deoxycholate, 0.2% SDS, and mixed protease inhibitors at 10^6^ cells/mL and was centrifuged (10 minutes at 16,000 *g*); and the soluble material was reserved as the DRM fraction.

### Sucrose density isolation

Neurons were harvested with a Teflon scraper and collected at 800 *g* for 5 minutes. Cell pellets were homogenized in a buffer containing 250 mM sucrose, 10 mM Tris-HCl (pH 7.4), 1 mM EGTA, mixed protease inhibitors, and 1 mM dithiothreitol at 10^6^ cells/mL. Particulate membrane fragments and nuclei were removed by centrifugation (1,000 *g* for 5 minutes). Membranes were washed by centrifugation at 16,000 *g* for 10 minutes at 4°C and suspended in an ice-cold buffer containing 1% Triton X-100, 10 mM Tris-HCl pH 7.2, 150 mM NaCl, 10 mM EDTA. Sucrose solutions of 5%, 10%, 15%, 20%, 25%, 30%, and 40% were prepared and layered over each other. Solubilized membranes were added on top and centrifuged at 50,000 *g* for 18 hours at 4°C. Serial 1-mL aliquots were collected from the bottom of gradients.

### Isolation of lysosomes

Lysosomes were isolated by using a lysosome enrichment kit designed for cultured cells in accordance with the instructions of the manufacturer (Pierce, Rockford, IL, USA). Briefly, treated cells were collected and homogenized in ice-cold lysosome enrichment reagents containing protease inhibitors (as above). The cell extract was overlaid on a discontinuous density gradient prepared from OptiPrep™ media and centrifuged at 145,000 *g* for 2 hours at 4°C. Serial 1-mL aliquots were collected from the bottom of gradients. The lysosome fraction was collected, washed three times in PBS (18,000 *g* for 30 minutes at 4°C), and solubilized in an extraction buffer containing 10 mM Tris-HCl pH 7.4, 100 mM NaCl, 10 mM EDTA, 0.5% Nonidet P-40, 0.5% sodium deoxycholate, 0.2% SDS, and protease inhibitors (as above). Samples were mixed with an equal volume of Laemmli buffer, boiled, and subjected to electrophoresis on a 15% polyacrylamide gel. Proteins were transferred onto a Hybond-P polyvinylidiene fluoride (PVDF) membrane by semi-dry blotting. Membranes were blocked by using 10% milk powder in PBS containing 0.2% Tween 20. Blots were probed with anti-lysosome-associated membrane protein 1 (LAMP-1) (Pharmingen, San Diego, CA, USA) followed by peroxidase-labelled anti-rabbit antibodies and visualized by using enhanced chemiluminescence.

### Isolation of endoplasmic reticulum

Endoplasmic reticulum (ER) fractions were isolated from treated cells by using an ER preparation kit (Sigma-Aldrich) in accordance with the instructions of the manufacturer. Homogenized cell extracts were separated on a discontinuous density gradient (OptiPrep™). Serial 1-mL aliquots were collected from the bottom of gradients. Fractions were diluted 1:20 in carbonate buffer and distributed into Nunc Maxisorp immunoplates (Nunc, Roskilde, Denmark) overnight. Plates were blocked with 5% milk powder and probed with monoclonal antibody (mAb) reactive to the ER marker Grp 78 (Stressgen Biotechnology, Victoria, Canada), polyclonal anti-PAF receptor (Cayman Chemical Company, Ann Arbor, MI, USA), anti-cholesterol ester hydrolase (CEH) (LifeSpan BioSciences, Seattle, WA, USA), or anti-acyl-coenzyme A:cholesterol acyltransferase (ACAT) (Abcam, Cambridge, UK), followed by biotinylated anti-rabbit IgG (Sigma-Aldrich), extravidin-alkaline phosphatase (Sigma-Aldrich), and the addition of the substrate p-nitrophenyl phosphate (p-NPP), 1 ng/mL, diluted in diethanolamine buffer. Optical density was read in a spectrophotometer at 405 nm.

### Cholesterol and protein content

Protein concentrations were measured by using a micro-BCA protein assay kit (Pierce). The amount of cholesterol was measured by using the Amplex Red cholesterol assay kit (Invitrogen, Carlsbad, CA, USA). Cholesterol was oxidized by cholesterol oxidase to yield hydrogen peroxide and ketones. The hydrogen peroxide reacts with 10-acetyl-3, 7-dihydroxyphenoxazine (Amplex Red) to produce highly fluorescent resorufin, which is measured by excitation at 550 nm and emission detection at 590 nm. Samples were incubated in the presence or absence of cholesterol esterase to determine the amounts of esterified cholesterol.

### Drugs

Arachidonyl trifluoromethyl ketone (AACOCF_3_), acetyl salicylic acid, diethylumbelliferyl phosphate (DEUP), ginkgolide B, ibuprofen, platelet-activating factor (PAF), 1-O-Hexadecyl-2-acetyl-*sn*-glycerol-3-phospho-(N,N,N-trimethyl)-hexanolamine (Hexa-PAF), methyl arachidonyl fluorophosphonate (MAFP), acetyl salicylic acid, ibuprofen, simvastatin, and squalene were all obtained from Sigma-Aldrich. Squalestatin was a gift from GlaxoSmithKline (Uxbridge, Middlesex, UK). Stock solutions were dissolved in ethanol or di-methyl sulphoxide and diluted in medium to obtain final working concentrations. Vehicle controls consisted of equivalent dilutions of ethanol or di-methyl sulphoxide in culture medium.

### Preparation of Aβ-containing medium

Conditioned media (CM) from Chinese hamster ovary (CHO) cells stably transfected with a cDNA encoding APP_751_ (referred to as 7PA2 cells) was cultured in Dulbecco’s modified Eagle’s medium with 10% fetal calf serum as described [15]. CM from these cells contains stable Aβ (7PA2-CM). 7PA2-CM was centrifuged at 100,000 *g* for 4 hours at 4°C to remove cell debris and then passed through a 50-kDa filter (Sartorius, Goettingen, Germany) before use. To demonstrate Aβ species, concentrated 7PA2-CM was mixed 1:1 with (0.5% NP-40, 5 mM CHAPS, 50 mM Tris-HCl, pH 7.4, and mixed protease inhibitors) and separated by polyacrylamide gel electrophoresis (PAGE) under non-denaturing conditions. Proteins were transferred onto a Hybond-P PVDF membrane (Amersham Biotech, now part of GE Healthcare, Little Chalfont, Buckinghamshire, UK) by semi-dry blotting. Membranes were blocked by using 10% milk powder and incubated with mAb 6E10 reactive with amino acids 1 to 16 of human Aβ (Signet, part of Covance, Princeton, NJ, USA). Bound mAbs were detected with biotinylated anti-mouse IgG, extravidin-peroxidase, and enhanced chemiluminescence.

### Size exclusion chromatography

7PA2-CM or brain extracts were concentrated by desalting columns (3-kDa filter; Sartorius) and injected into a Superdex 75 PC column (separates peptides ranging from 3 to 70 kDa) (GE Healthcare) and eluted at a rate of 0.2 mL/minute. Fractions were collected and tested by Aβ enzyme-linked immunosorbent assay (ELISA) (see below).

### Sample preparation

To detach Aβ_42_ from cellular binding proteins that could occlude specific epitopes, cell extracts (200 μL) were mixed with 500 μL of 70% formic acid and sonicated. A 50-μL aliquot was added to 450 μL of 1 M Tris-HCl with protease inhibitors (as above) and sonicated before addition to ELISA.

### Synthetic Aβ

A peptide corresponding to amino acids 1 to 42 of Aβ (Aβ_1-42_) was obtained from Bachem (Bubendorf, Switzerland) and dissolved in hexafluoroisopropanol, lyophilized, and stored at −80°C. On the day of use, it was dissolved in dimethylsulphoxide at 1 mM, diluted in culture medium, and sonicated prior to addition to cells.

### Aβ_42_ enzyme-linked immunosorbent assay

Nunc Maxisorp immunoplates were coated with mAb 4G8 (epitope 17-24) (Covance) in carbonate buffer overnight. Plates were blocked with 5% milk powder in PBS-tween, and samples were applied. The detection antibody was an Aβ_42_ selective rabbit mAb BA3-9 (Covance) followed by biotinylated anti-rabbit IgG (Sigma-Aldrich). Total Aβ was visualized by addition of the substrate (pNPP) 1 ng/mL, diluted in diethanolamine buffer, and optical density was read in a spectrophotometer at 405 nm.

### Statistical methods

Differences between treatment groups were assessed by using Student *t* tests.

## Results

### Squalestatin increased the degradation of Aβ_42_

**Figure 1:**
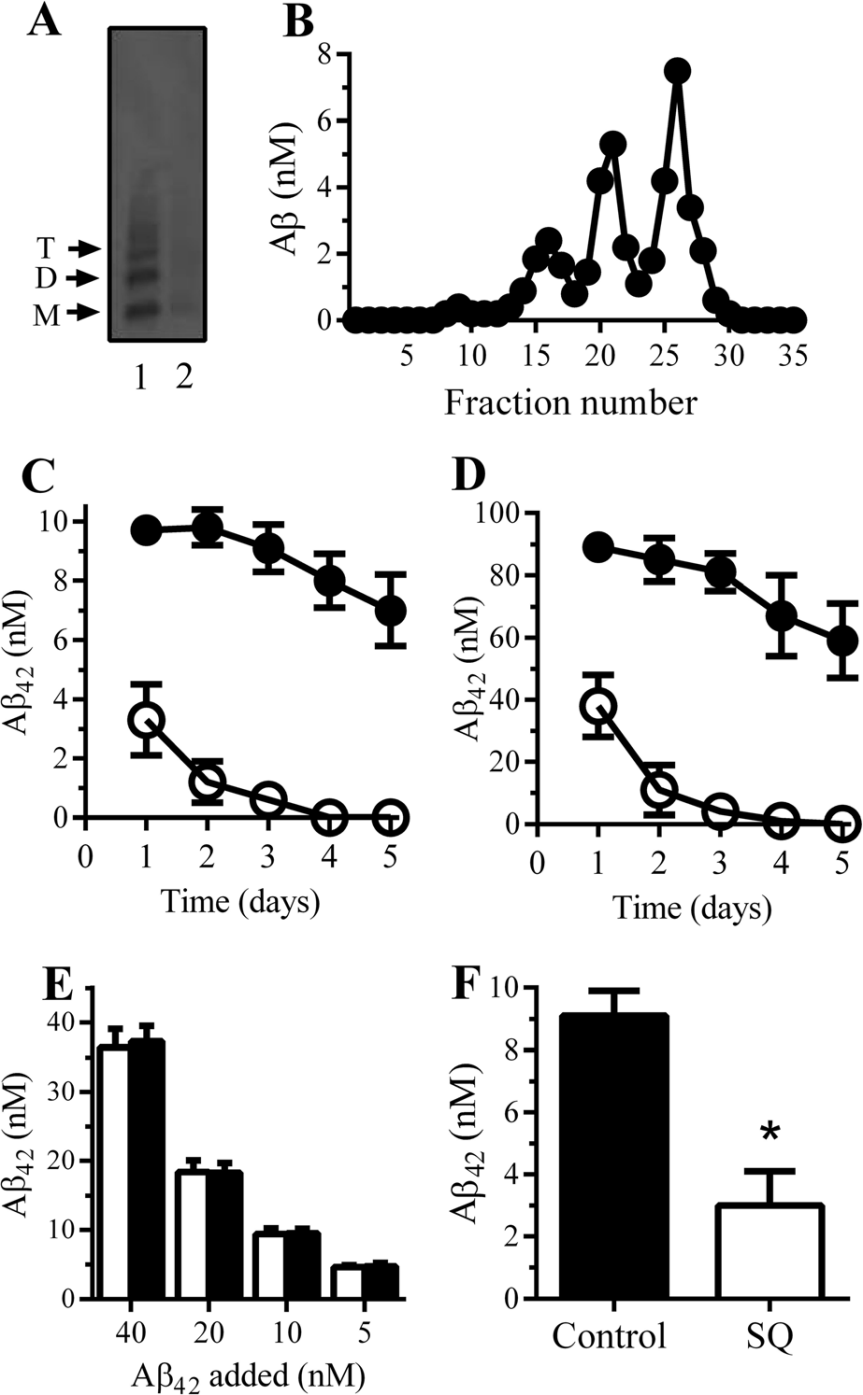
**Squalestatin increased the degradation of Aβ**_**42**_**. (A)** Immunoblot showing amyloid-beta (Aβ) monomers (M), dimers (D), and trimers (T) in conditioned media from 7PA2 cells (7PA2-CM) (1) and in Aβ-depleted 7PA2-CM (2). **(B)** Aβ monomers, dimers, and trimers eluted from a Superdex 75 PC column (separates proteins ranging from 3 to 70 kDa) loaded with concentrated 7PA2-CM. Values are mean Aβ_42_ of duplicates. **(C)** Neurons were pre-treated with control medium (●) or 200 nM squalestatin (○), pulsed with 7PA2-CM containing 10 nM Aβ_42_, and incubated for 1 to 5 days as shown. Values are mean Aβ_42_ remaining in neurons ± standard deviation (SD) from triplicate experiments performed four times (n = 12). **(D)** Neurons were pre-treated with control medium (●) or 200 nM squalestatin (○), pulsed with 100 nM synthetic Aβ_42_, and incubated for 1 to 5 days as shown. Values are mean Aβ_42_ in neurons ± SD from triplicate experiments performed four times (n = 12). **(E)** Neurons were pre-treated with control medium (■) or 200 nM squalestatin (□) and pulsed with 7PA2-CM containing Aβ_42_ as shown for 2 hours. Values are mean neuronal Aβ_42_ ± SD from triplicate experiments performed three times (n = 9). **(F)** Neurons, incubated with 7PA2-CM containing 10 nM Aβ_42_ for 2 days, were treated with control medium (■) or 200 nM squalestatin (□) for 24 hours. Values are the mean Aβ_42_ remaining in neurons ± SD from triplicate experiments performed three times (n = 9). *Concentrations of Aβ_42_ significantly less (*P* <0.05) than controls.

The amount and distribution of cholesterol are recognized as factors that affect the progression of AD [29,30]. As squalestatin reduced the cholesterol content of cultured neurons [31], the fate of Aβ_42_ in squalestatin-treated neurons was examined. Treatment with 200 nM squalestatin for 24 hours reduced the cholesterol content of neurons from 519 ng cholesterol/10^6^ cells ± 48 to 369 ng ± 66, n = 6, *P* <0.05, without affecting neuronal viability as measured by thiazolyl blue tetrazolium (MTT). Both treated and untreated neurons were subsequently incubated with 7PA2-CM containing 10 nM Aβ_42_, a mixture of monomers, dimers, trimers, and tetramers as shown in Figure 1A. An analysis of concentrated 7PA2-CM by size exclusion chromatography also showed that it contained three main peaks corresponding to monomers, dimers, and trimers (Figure 1B). The concentrations of Aβ_42_ used did not affect neuronal survival during the 5-day incubation period. In control neurons, Aβ_42_ had a half-life of greater than 5 days, whereas in squalestatin-treated neurons, the half-life of Aβ_42_ was reduced to less than 24 hours (Figure 1C). Aβ_42_ was not detected in cell supernatants, suggesting that the Aβ_42_ had been digested rather than secreted from neurons. Experiments performed using synthetic Aβ_1-42_ showed that it behaved like the cell-derived Aβ_42_; it had a half-life of greater than 5 days in untreated neurons and a much shorter half-life in squalestatin-treated neurons (Figure 1D).

Similar results were obtained in neurons that had been treated with 1 μM simvastatin, a clinically approved hydroxymethyl glutaryl coenzyme A reductase inhibitor that also reduced cellular cholesterol levels. As cholesterol depletion has been demonstrated to affect the expression of some receptors, including the cellular prion protein [32] and acetylcholine receptors [33], the possibility that it reduced the binding of Aβ_42_ to neurons was explored. There were no significant differences in the amounts of cell-bound Aβ_42_ between control and squalestatin-treated neurons, indicating that cholesterol depletion did not affect the binding of Aβ_42_ to neurons (Figure 1E). In further studies, neurons were pulsed with 10 nM Aβ_42_; after 2 days, 200 nM squalestatin was added, and cell extracts were collected 1 day later. The amount of Aβ_42_ in squalestatin-treated neurons was significantly less than in mock-treated neurons (Figure 1F).

### Squalestatin altered the distribution of Aβ_42_ in neurons

**Figure 2:**
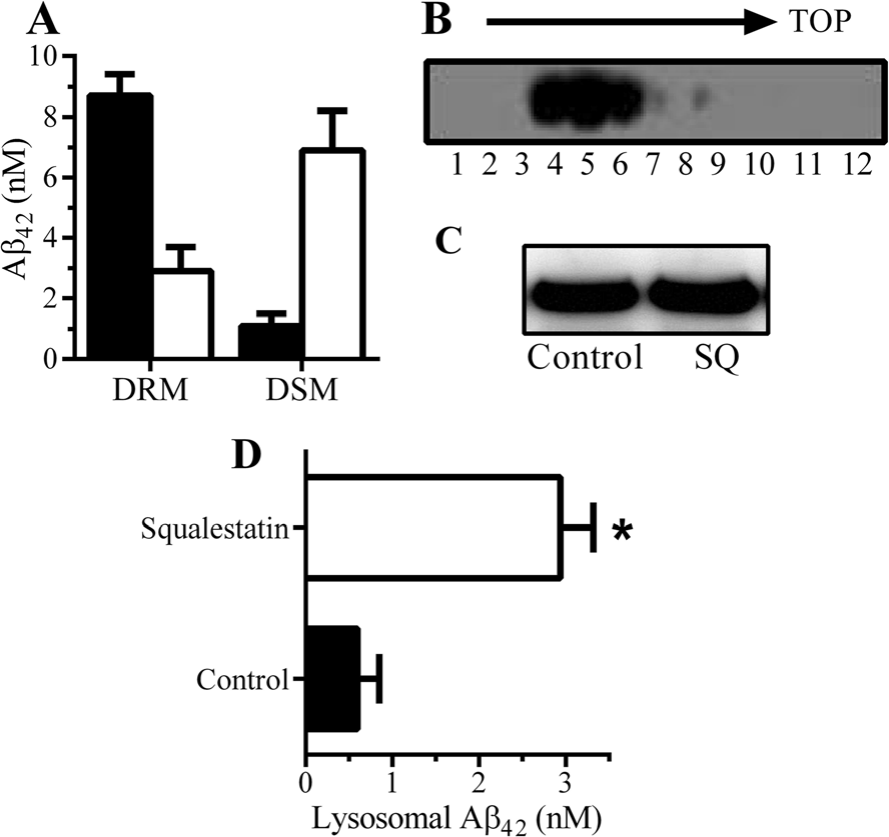
**Squalestatin alters the cellular distribution of Aβ**_**42**_**. (A)** Concentrations of Aβ_42_ in detergent-resistant membranes (DRMs) (rafts) or detergent-soluble membranes (DSMs) 2 hours after the addition of conditioned media from 7PA2 cells (7PA2-CM) containing 10 nM Aβ_42_ to neurons pre-treated with control medium (■) or 200 nM squalestatin (□). Values are mean ± standard deviation (SD) from triplicate experiments performed three times (n = 9). **(B)** Cell extracts from neurons were separated on sucrose density gradients and analyzed by immunoblot for lysosome-associated membrane protein 1 (LAMP-1), a marker of lysosomes. **(C)** Immunoblot showing the amount of LAMP-1 in extracts from neurons treated with control medium or 200 nM squalestatin. **(D)** The amount of Aβ_42_ in lysosomes derived from neurons treated with control medium (□) or 200 nM squalestatin (□) and incubated with 7PA2-CM containing 10 nM Aβ_42_ for 2 hours. Values are mean ± SD from triplicate experiments performed three times (n = 9). *Concentrations of Aβ_42_ significantly higher (*P* <0.05) than controls.

Next, we looked at the distribution of Aβ_42_ within neurons. In control neurons incubated with 10 nM Aβ_42_ for 2 hours, most Aβ_42_ was found within DRMs, observations that are consistent with reports that membrane-bound Aβ was recruited into lipid rafts [19-21,34,35]. In squalestatin-treated neurons, significantly less Aβ_42_ was found within DRMs (Figure 2A). Since many of the proteins that are targeted to lipid rafts traffic via a recycling pathway that avoids the lysosomes [23,36], an organelle fractionation technique was used to examine the targeting of Aβ_42_ to lysosomes. Organelles from neurons were separated on a density gradient, and lysosomes were identified by using immunoblots for lysosome-associated antigen 1 (LAMP-1), a marker of late endosomes/lysosomes. LAMP-1 was enriched within fractions 4 to 6, which were subsequently pooled to represent the lysosomes (Figure 2B). Firstly, we showed that treatment with 200 nM squalestatin did not affect the amount of the LAMP-1 found in neurons (Figure 2C). Next, treated neurons were incubated with 10 nM Aβ_42_ for 2 hours, and the amount of Aβ_42_ in lysosomes was measured. The amounts of Aβ_42_ in lysosomes from squalestatin-treated neurons were significantly higher than the amounts of Aβ_42_ in lysosomes from control neurons (Figure 2D).

### Squalene reversed the effect of squalestatin on the cellular distribution of Aβ_42_

One explanation of these results is that the squalestatin-induced reduction in cholesterol disrupts the formation of lipid rafts that direct Aβ_42_ into a pathway that avoids the lysosomes. This hypothesis was tested by incubating neurons with combinations of squalestatin and squalene. The addition of 5 μM squalene alone did not increase cholesterol concentrations above those of control cells (527 ng cholesterol/10^6^ cells ± 28 to 519 ng ± 48, n = 6, *P* = 0.74), but the addition of 5 μM squalene reversed the effects of 200 nM squalestatin on cellular cholesterol concentrations (496 ng cholesterol/10^6^ cells ± 45 to 369 ng ± 66, n = 6, *P* <0.05). The addition of 5 μM squalene also reversed the effects of 200 nM squalestatin on the trafficking of Aβ_42_; the amount of Aβ_42_ in DRMs was significantly lower and the amount in lysosomes significantly higher in neurons treated with squalestatin when compared with neurons treated with a combination of squalestatin and squalene (Table 1). Furthermore, the effect of squalestatin on the degradation of Aβ_42_ by neurons was reversed by the addition of squalene. When neurons incubated with 10 nM Aβ_42_ for 2 days were subsequently treated with combinations of squalestatin and squalene for 24 hours, the amounts of Aβ_42_ in neurons treated with 200 nM squalestatin and 5 μM squalene were significantly higher than in neurons treated with 200 nM squalestatin alone (8.9 nM Aβ_42_ ± 0.8 compared with 2.4 nM Aβ_42_ ± 0.6, n = 9, *P* <0.01).

**Table 1:** **Squalene reversed the effects of squalestatin upon Aβ**_**42 **_**trafficking**

	**Concentration of Aβ**_**42**_**, nM**		
	**Cell extracts**	**DRMs**	**Lysosomes**
**Control**	8.7 ± 0.7	8.4 ± 0.6	0.4 ± 0.2
**Squalestatin**	8.7 ± 1	2.4 ± 0.6^a^	2.8 ± 0.5^b^
**Squalene**	8.5 ± 0.85	8 ± 0.4	0.5 ± 0.2
**Squalestatin + squalene**	8.3 ± 0.6	7.9 ± 0.9	0.3 ± 0.1

Neurons were pre-treated with 200 nM squalestatin, 5 μM squalene, or a mixture of squalestatin and squalene and then incubated with conditioned media from 7PA2 cells (7PA2-CM) containing 10 nM Aβ_42_ for 2 hours. The concentrations of Aβ_42_ in whole cell extracts, detergent-resistant membranes (DRMs) (lipid rafts), and lysosomes were measured by enzyme-linked immunosorbent assay. Values are the mean concentration of Aβ_42_ ± standard deviation from triplicate experiments performed three times (n = 9). ^a^Amounts of Aβ_42_ significantly lower than in control cells. ^b^Amounts of Aβ_42_ significantly higher than in control cells.

### Phospholipase A_2_ and platelet-activating factor affect the degradation of Aβ_42_

**Figure 3:**
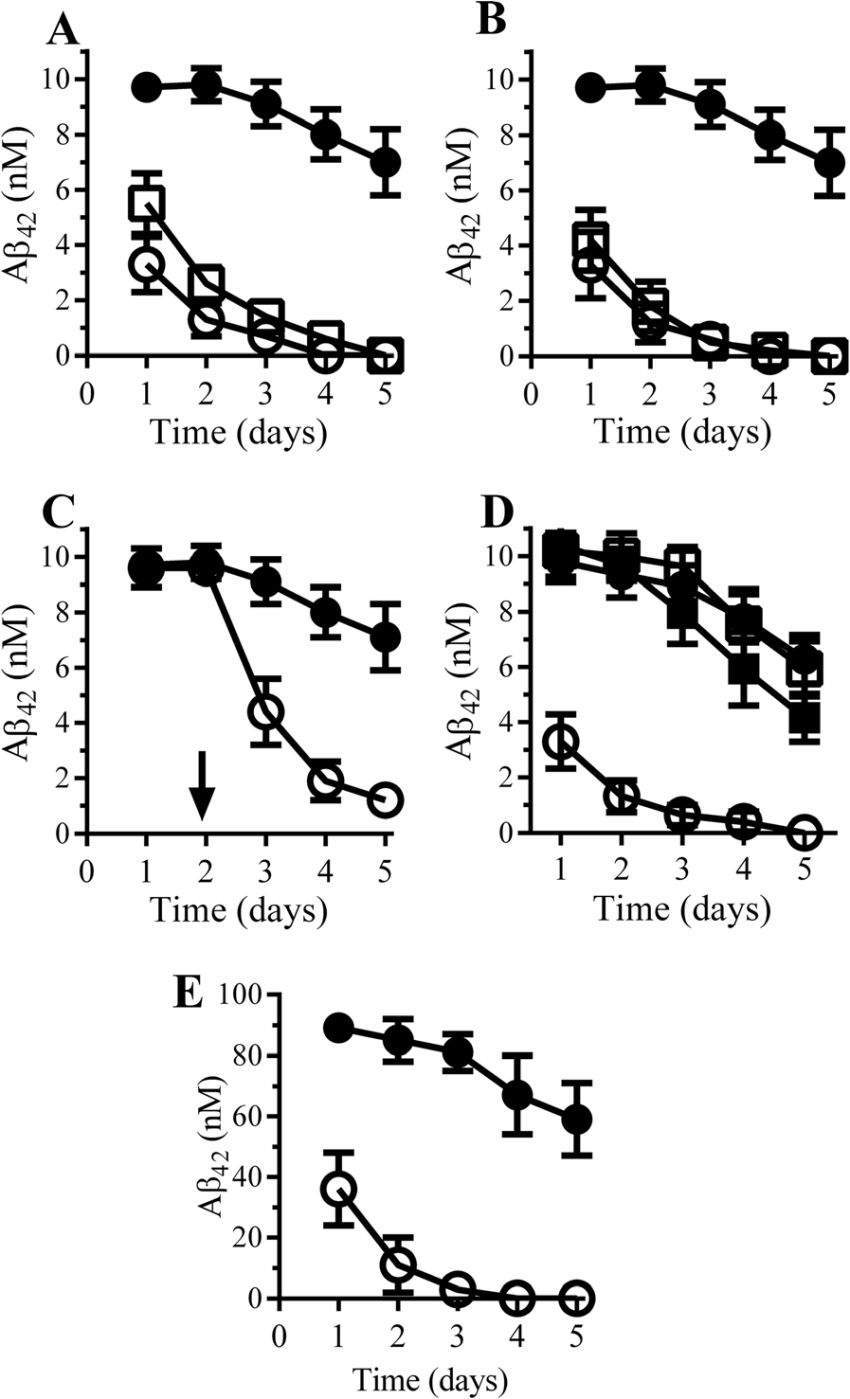
**Platelet-activating factor (PAF) receptor antagonists increased the degradation of Aβ**_**42**_**. (A)** Amounts of Aβ_42_ in neurons pre-treated with control medium (●), 1 μM AACOCF_3_ (○), or 1 μM MAFP (□), pulsed with conditioned media from 7PA2 cells (7PA2-CM) containing 10 nM Aβ_42_, and incubated for 1 to 5 days. Values are mean ± standard deviation (SD) from triplicate experiments performed four times (n = 12). **(B)** Amounts of Aβ_42_ in neurons pre-treated with control medium (●), 1 μM Hexa-PAF (○), or 1 μM ginkgolide B (□), pulsed with 7PA2-CM containing 10 nM Aβ_42_, and incubated for 1 to 5 days. Values are mean ± SD from triplicate experiments performed four times (n = 12). **(C)** Neurons were incubated with 7PA2-CM containing 10 nM Aβ_42_. After 2 days, 1 μM Hexa-PAF (○) or control medium (●) was added and the amounts of Aβ_42_ were measured thereafter. Values are mean Aβ_42_ ± SD from triplicate experiments performed four times (n = 12). **(D)** Neurons were pre-treated with control medium (●), 1 μM PAF (□), 1 μM AACOCF_3_ (○), or a combination of 1 μM AACOCF_3_ and 1 μM PAF (■) and incubated with 7PA2-CM containing 10 nM Aβ_42_ for 1 to 5 days. Values are mean Aβ_42_ ± SD from triplicate experiments performed three times (n = 9). **(E)** The amount of Aβ_42_ in neurons pre-treated with control medium (●) or 1 μM Hexa-PAF (○), pulsed with 100 nM synthetic Aβ_42_, and incubated for 1 to 5 days. Values are mean ± SD, from triplicate experiments performed four times (n = 12). AACOCF_3_, arachidonyl trifluoromethyl ketone; Hexa-PAF, 1-O-hexadecyl-2-acetyl-*sn*-glycerol-3-phospho-(N,N,N-trimethyl)-hexanolamine; MAFP, methyl arachidonyl fluorophosphonate.

As Aβ activates cytoplasmic phospholipase A_2_ (cPLA_2_) [37-40], an enzyme that is implicated in tubule formation and intracellular trafficking [41-43], specifically within the trans-Golgi network [44], the effects of cPLA_2_ inhibitors on Aβ_42_ distribution were studied. Neurons were pulsed with 7PA2-CM containing 10 nM Aβ_42_ for 2 hours and were washed, and cell extracts were collected at time points thereafter. Pre-treatment of neurons with cPLA_2_ inhibitors (1 μM AACOCF_3_ or MAFP) significantly reduced the half-life of Aβ_42_ in neurons (Figure 3A). As the activation of cPLA_2_ results in the production of prostaglandins and PAF, specific inhibitors/antagonists of these mediators were also tested. The half-life of Aβ_42_ was reduced in neurons pre-treated with PAF receptor antagonists (1 μM ginkgolide B or 1 μM Hexa-PAF) (Figure 3B) but not in neurons pre-treated with cyclo-oxygenase inhibitors (1 μM aspirin or 2 μM ibuprofen) that reduce the production of prostaglandins. In neurons pre-treated with ginkgolide B or Hexa-PAF, the half-life of Aβ_42_ was reduced to less than 24 hours. The addition of 1 μM Hexa-PAF to neurons that had been incubated with 10 nM Aβ_42_ for 2 days resulted in the increased degradation of Aβ_42_ (Figure 3C). Aβ_42_ was not detected in the supernatants collected from either control or treated neurons, indicating that it was not secreted. Such results suggest that PAF, produced in response to Aβ_42_-induced activation of cPLA_2_, regulates the trafficking and hence the degradation of Aβ_42_ in neurons. In support of this hypothesis, we found that the addition of 1 μM PAF reversed the effects of the cPLA_2_ inhibitor (AACOCF_3_) upon the degradation of Aβ_42_ in neurons (Figure 3D). Experiments performed using synthetic Aβ_1-42_ showed that it had a half-life of approximately 24 hours in neurons pre-treated with Hexa-PAF (Figure 3E).

### Hexa-PAF altered the distribution of Aβ_42_ in neurons

**Figure 4:**
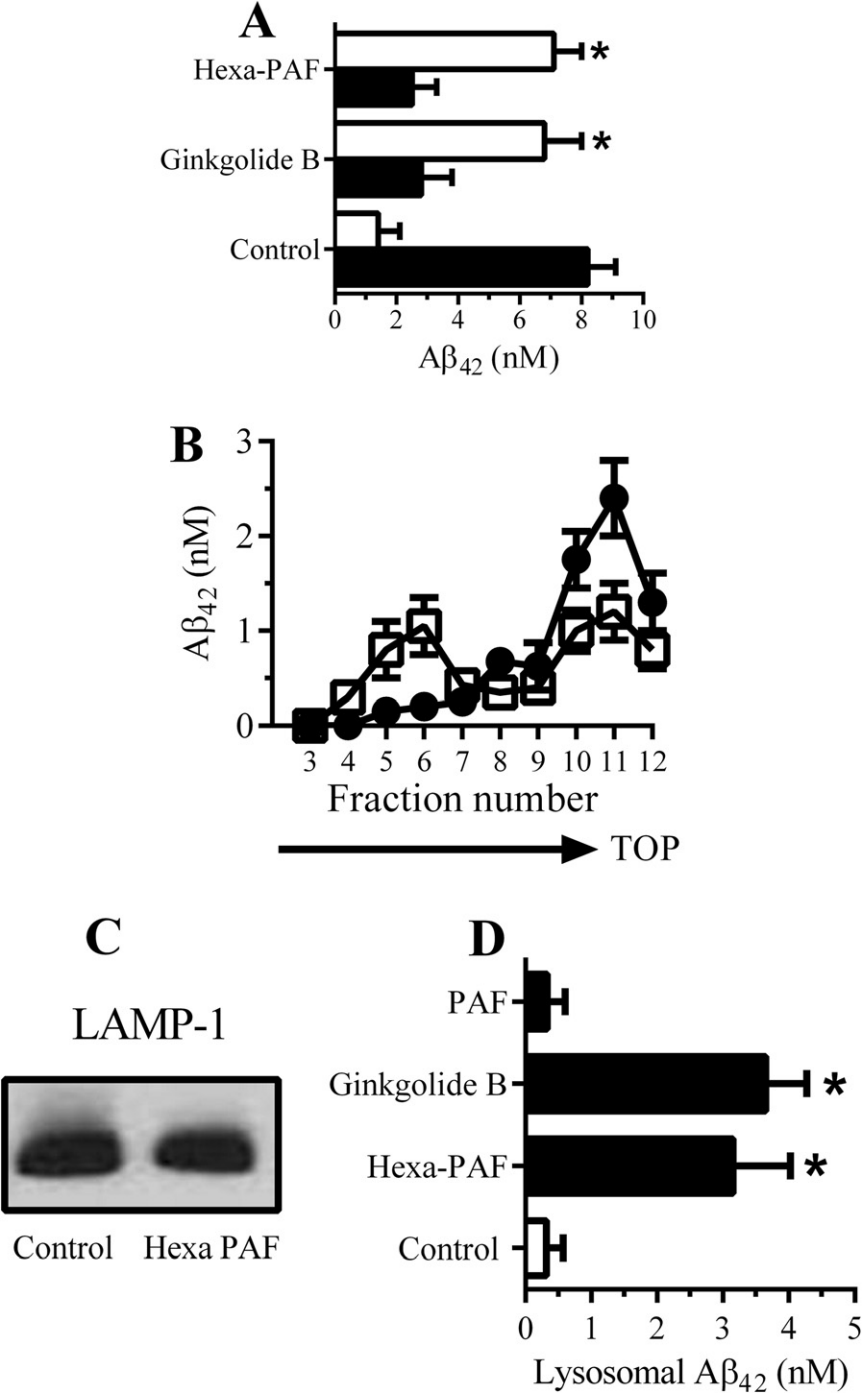
**Platelet-activating factor (PAF) receptor antagonists altered the distribution of Aβ**_**42 **_**in neurons. (A)** The amount of Aβ_42_ in detergent-soluble membrane (DSM) (□) or detergent-resistant membrane (DRM) (■) extracts from neurons pre-treated with control medium, 1 μM ginkgolide B, or 1 μM Hexa-PAF and pulsed with conditioned media from 7PA2 cells (7PA2-CM) containing 10 nM Aβ_42_ for 2 hours. Values are mean ± standard deviation (SD) from triplicate experiments performed four times (n = 12). *Concentration of Aβ_42_ significantly higher (*P* <0.05) than in control neurons. **(B)** Neurons treated with control medium (●) or 1 μM Hexa-PAF (□) and pulsed with 7PA2-CM containing 10 nM Aβ_42_ for 2 hours. Cell extracts were separated on sucrose density gradients. Values are mean Aβ_42_ ± SD from triplicate experiments (n = 3). **(C)** Immunoblot showing the amounts of lysosome-associated membrane protein 1 (LAMP-1) in neurons treated with control medium or 1 μM Hexa-PAF. **(D)** Neurons were pre-treated with control medium (□), 1 μM Hexa-PAF, 1 μM ginkgolide B, or 1 μM PAF and pulsed with 7PA2-CM containing 10 nM Aβ_42_ for 2 hours when lysosomes were isolated. Values are mean Aβ_42_ ± SD from triplicate experiments performed four times (n = 12). *Concentration of Aβ_42_ significantly higher (*P* <0.05) than in control neurons. Hexa-PAF, 1-O-hexadecyl-2-acetyl-*sn*-glycerol-3-phospho-(N,N,N-trimethyl)-hexanolamine.

As the effects of Hexa-PAF on the neuronal degradation of Aβ_42_ were similar to those of squalestatin, its effects upon cellular distribution of Aβ_42_ were studied. After the addition of 10 nM Aβ_42_ for 2 hours, we found no significant differences in the amounts of cell-associated Aβ_42_ between control and Hexa-PAF-treated neurons (9.1 nM Aβ_42_ ± 0.7 compared with 8.7 ± 1, n = 9, *P* = 0.32). However, pre-treatment of neurons with 1 μM Hexa-PAF or 1 μM ginkgolide B significantly reduced the amount of Aβ_42_ found within DRMs (Figure 4A). Since the detergent solubility assay is a crude test of membrane targeting, neurons were incubated for 2 hours with 10 nM Aβ_42_ and membranes were separated on sucrose density gradients. In control neurons, most of the Aβ_42_ was found in low-density membrane fractions. In neurons pre-treated with 1 μM Hexa-PAF, less of the Aβ_42_ was found within low-density fractions (Figure 4B). Although treatment with 1 μM Hexa-PAF did not affect the amount of the LAMP-1 found in neurons (Figure 4C), the amounts of Aβ_42_ in lysosomes from neurons pre-treated with Hexa-PAF or ginkgolide B were significantly higher than the amounts of Aβ_42_ in lysosomes from control neurons (Figure 4D).

### The effects of Hexa-PAF are not reversed by squalene

**Figure 5:**
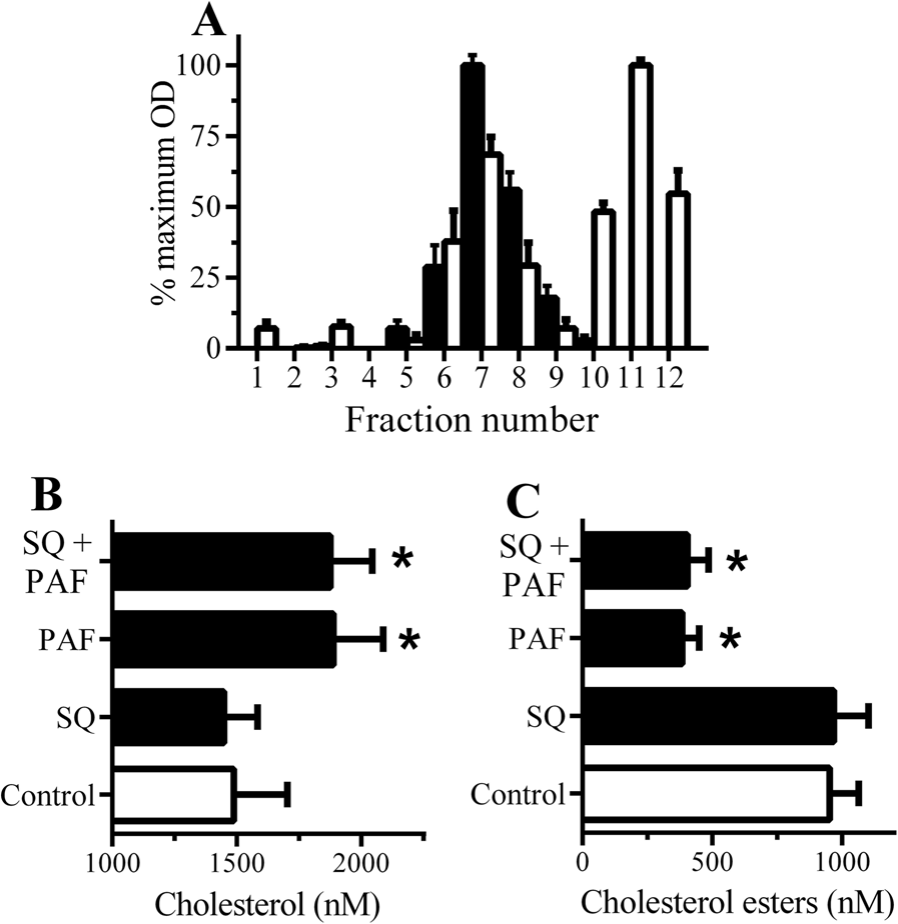
**Platelet-activating factor (PAF) increased cholesterol in the endoplasmic reticulum (ER) of neurons. (A)** Neuronal extracts were separated by density gradient centrifugation and analyzed for PAF receptor (□) and the ER marker Grp 78 (■). **(B)** The amounts of cholesterol in ER fractions incubated with control medium (□), 1 μM squalestatin, or 2 μM PAF or pre-treated with squalestatin and incubated with PAF (■) for 1 hour. Values are mean ± standard deviation (SD) from triplicate experiments performed three times (n = 9). *Concentration of cholesterol significantly higher (*P* <0.05) than in controls. **(C)** The amounts of cholesterol esters in ER fractions incubated with control medium (□), 1 μM squalestatin, or 2 μM PAF or pre-treated with squalestatin and incubated with PAF (■). Values are mean ± SD from triplicate experiments performed three times (n = 9). *Concentration of cholesterol esters significantly lower (*P* <0.05) than in controls.

The similar effects of squalestatin and Hexa-PAF upon Aβ_42_ trafficking suggested that Hexa-PAF may affect cholesterol synthesis. However, treatment with either 1 μM PAF or 1 μM Hexa-PAF did not significantly affect the amounts of cholesterol in neurons and the addition of 5 μM squalene did not reverse the effects of Hexa-PAF upon the targeting of Aβ_42_ to DRMs or lysosomes (Table 2) nor alter the half-life of Aβ_42_ in Hexa-PAF-treated neurons, indicating that Hexa-PAF did not affect cholesterol synthesis. Next, we explored the hypothesis that PAF increased cholesterol in specific cellular compartments. We sought to identify such compartments by separating organelles upon density gradients. PAF receptors were found in high concentrations in fractions 6 to 8 (Figure 5A), fractions that also contained Grp78, a marker of the ER. These fractions were subsequently pooled and incubated with or without PAF. The addition of 2 μM PAF caused a significant increase in cholesterol (Figure 5B). The PAF-induced increase in cholesterol in these fractions was not blocked by the inclusion of 200 nM squalestatin, indicating that PAF was not stimulating cholesterol synthesis. Conversely, the addition of PAF significantly reduced the amounts of cholesterol esters in ER fractions (Figure 5C).

**Table 2:** **Squalene does not reverse the effects of Hexa-PAF upon Aβ**_**42 **_**trafficking**

	**Concentration of Aβ**_**42**_**, nM**		
	**Cell extracts**	**DRMs**	**Lysosomes**
**Control**	8.7 ± 0.7	8.4 ± 0.6	0.4 ± 0.2
**Hexa-PAF**	8.5 ± 1.2	2.5 ± 0.9^a^	3 ± 0.6^b^
**Squalene**	8.5 ± 0.9	8 ± 0.4	0.5 ± 0.2
**Hexa-PAF + squalene**	8.8 ± 0.6	2.8 ± 0.8^a^	3 ± 0.4^b^

Neurons were pre-treated with 1 μM Hexa-PAF, 5 μM squalene, or a mixture of Hexa-PAF and squalene and then incubated with conditioned media from 7PA2 cells (7PA2-CM) containing 10 nM Aβ_42_ for 2 hours. The amounts of Aβ_42_ in whole cell extracts, detergent-resistant membranes (DRMs) (lipid rafts), and lysosomes were measured by enzyme-linked immunosorbent assay. Values are the mean concentration of Aβ_42_ ± standard deviation from triplicate experiments performed three times (n = 9). ^a^Amounts of Aβ_42_ significantly lower than in control cells. ^b^Amounts of Aβ_42_ significantly higher than in control cells. Hexa-PAF, 1-O-hexadecyl-2-acetyl-*sn*-glycerol-3-phospho-(N,N,N-trimethyl)-hexanolamine.

### Platelet-activating factor stimulates the release of cholesterol from stores of cholesterol esters

**Figure 6:**
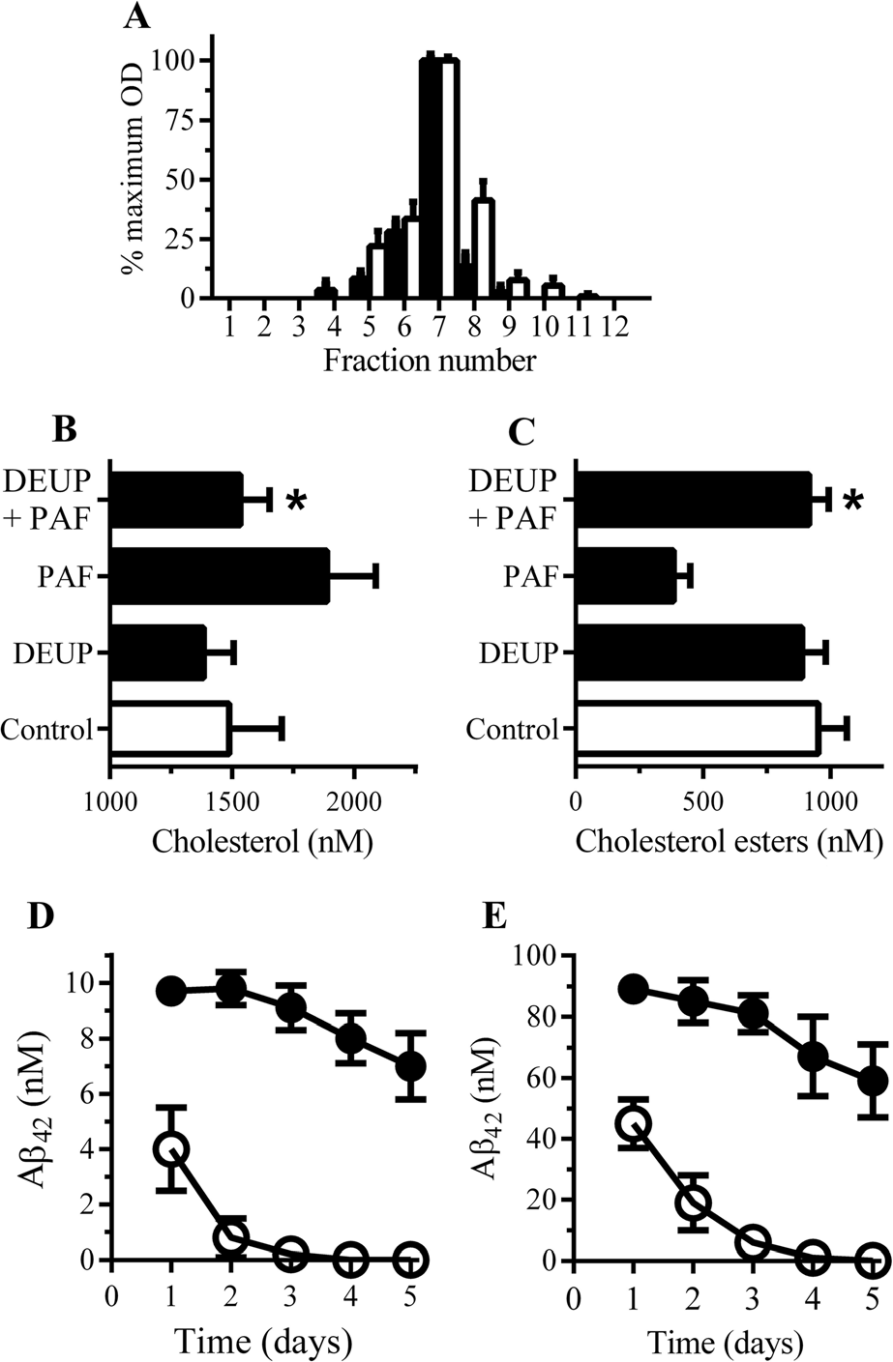
**Platelet-activating factor (PAF) causes the release of cholesterol in the endoplasmic reticulum (ER). (A)** Neuronal extracts were separated by density gradient centrifugation and analyzed for cholesterol ester hydrolase (CEH) (□) and anti-acyl-coenzyme A:cholesterol acyltransferase (ACAT) (■). **(B)** The amount of cholesterol in ER fractions pre-treated with control medium (□), 20 μM diethylumbelliferyl phosphate (DEUP), or 2 μM PAF or pre-treated with DEUP and incubated with PAF (■). Values are mean ± standard deviation (SD) from triplicate experiments performed three times (n = 9). *Significantly less (*P* <0.05) cholesterol than ER fractions incubated with PAF. **(C)** The amount of cholesterol esters in ER fractions incubated with control medium (□), 20 μM DEUP, or 2 μM PAF or pre-treated with DEUP and incubated with PAF (■). Values are mean ± SD from triplicate experiments performed three times (n = 9). *Significantly more (*P* <0.05) cholesterol esters than ER fractions incubated with PAF. **(D)** Neurons treated with control medium (●) or 20 μM DEUP (○) were pulsed with conditioned media from 7PA2 cells (7PA2-CM) containing 10 nM Aβ_42_ and incubated for 1 to 5 days as shown. Values are mean Aβ_42_ ± SD from triplicate experiments performed four times (n = 12). **(E)** The amount of Aβ_42_ in neurons pre-treated with control medium (●) or 20 μM DEUP (○), pulsed with 100 nM synthetic Aβ_42_, and incubated for 1 to 5 days. Values are mean Aβ_42_ ± SD from triplicate experiments performed four times (n = 12).

To determine the source of the PAF-induced increase in cholesterol, other methods of cholesterol regulation were explored. The amounts of cholesterol within cells is tightly controlled via a mixture of synthesis, uptake, and efflux and by esterification of cholesterol [45]. The cholesterol ester cycle involves the esterification of cholesterol by ACAT and the storage of cholesterol esters as cytoplasmic droplets [46]. Conversely, the hydrolysis of cholesterol esters results in the release of cholesterol from these stores. Thus, the cholesterol ester cycle helps maintain a constant level of cholesterol in cell membranes [46,47]. Our findings that that both ACAT and CEH were concentrated in the ER fractions along with PAF receptors (Figure 6A) suggested that PAF-induced activation of CEH released cholesterol. To explore the role of CEH in the trafficking of Aβ_42_, an inhibitor of CEH (DEUP) was used [48]. Treatment with 20 μM DEUP did not significantly affect total neuronal cholesterol levels (552 ng cholesterol ± 34 compared with 519 ng ± 48, n = 6, *P* = 0.26). However, pre-treatment of ER fractions with 20 μM DEUP blocked the PAF-induced increase in cholesterol (Figure 6B) and the PAF-induced reduction in cholesterol esters (Figure 6C). Pre-treatment of neurons with 20 μM DEUP did not alter the amount of Aβ_42_ that bound to neurons, but reduced the amounts of Aβ_42_ in DRMs and increased the amounts of Aβ_42_ in lysosomes (Table 3). As a consequence, the half-life of Aβ_42_ in DEUP-treated cells was reduced to approximately 24 hours (Figure 6D). Pre-treatment of neurons with 20 μM DEUP also reduced the half-life of synthetic Aβ_1-42_ to approximately 24 hours (Figure 6E). Notably, these effects of DEUP were not reversed by the inclusion of 5 μM squalene, indicating that they were not mediated by inhibition of cholesterol synthesis. Collectively these results suggest that CEH regulates the hydrolysis of cholesterol esters in the ER which is involved in the trafficking of Aβ_42_.

**Table 3:** **Squalene does not reverse the effects of DEUP upon Aβ**_**42 **_**trafficking**

	**Concentration of Aβ**_**42**_**, nM**		
	**Cell extracts**	**DRMs**	**Lysosomes**
**Control**	8.7 ± 0.7	8.4 ± 0.6	0.4 ± 0.2
**DEUP**	8.6 ± 0.9	2.2 ± 0.7^a^	2.8 ± 0.3^b^
**Squalene**	8.5 ± 0.9	8 ± 0.4	0.5 ± 0.2
**DEUP + squalene**	8.9 ± 0.8	2 ± 0.6^a^	2.7 ± 0.3^b^

Neurons were pre-treated with 20 μM diethylumbelliferyl phosphate (DEUP), 5 μM squalene, or a mixture of DEUP and squalene and then incubated with conditioned media from 7PA2 cells (7PA2-CM) containing 10 nM Aβ_42_ for 2 hours. The amounts of Aβ_42_ in whole cell extracts, detergent-resistant membranes (DRMs) (lipid rafts), and lysosomes were measured by enzyme-linked immunosorbent assay. Values are the mean concentration of Aβ_42_ ± standard deviation from triplicate experiments performed three times (n = 9). ^a^Amounts of Aβ_42_ significantly less than in control cells. ^b^Amounts of Aβ_42_ significantly higher than in control cells.

## Discussion

This study showed that Aβ_42_ taken up by cultured neurons was found predominantly within lipid rafts, traffics via a pathway that avoids the lysosomes and consequently was cleared slowly. We hypothesize that the slow clearance of Aβ_42_ from neurons is a significant factor in the accumulation of Aβ_42_ within the brain and leads to synapse damage and dementia in AD. Critically we show that these properties are dependent upon Aβ_42_-induced activation of PLA_2_, the production of PAF, and the activation of CEH, the blockade of which alters the distribution of Aβ_42_ in neurons and greatly increased its degradation.

This study used nanomolar concentrations of Aβ which did not affect the viability of neurons and which were similar to those found in the cerebrospinal fluid [49,50] or in soluble brain extracts from patients with AD [51-53]. In control neurons, most of the Aβ was found within rafts, an observation consistent with reports that Aβ in the brain is found within cholesterol-dense membrane rafts [19-21]. Reports that Aβ affects cellular cholesterol homeostasis [54-56] suggested that Aβ modifies the biochemistry of cell membranes and thus creates the rafts that are responsible for its trafficking. The targeting of Aβ_42_ to rafts may have important biological consequences as many of the proteins that are targeted to rafts avoid the classic endocytic trafficking pathway that ends in the lysosomes [22,57]. Our observations were compatible with Aβ_42_ trafficking via a recycling pathway in neurons as little Aβ_42_ was found within lysosomes and consequently Aβ_42_ had a long half-life (over 5 days) in these neuronal cultures.

Here, we show that the trafficking of Aβ_42_ within neurons could be affected by inhibiting cholesterol synthesis. Cholesterol has multiple effects upon cells, including altering membrane fluidity, protein trafficking, endocytosis, and exocytosis [58]. More specifically, cholesterol is important for the formation and function of membrane rafts, and in cholesterol-depleted neurons, most of the Aβ_42_ was excluded from such rafts. Consequently, more Aβ_42_ was found within lysosomes and the half-life of Aβ_42_ was reduced from over 5 days in untreated neurons to less than 24 hours in squalestatin-treated neurons. Squalestatin was used because it inhibits squalene synthetase and reduces cholesterol production without affecting the production of the isoprenoids [27]. However, because squalestatin does not cross the blood-brain barrier, it does not have clinical application. Similar results were obtained in neurons treated with simvastatin, a cholesterol synthesis inhibitor that crosses the blood-brain barrier (data not shown). Simvastatin is widely used clinically and has been reported to reduce cholesterol levels within the brain [59,60].

Since cholesterol synthesis inhibitors affect many cholesterol-sensitive processes, we sought to find a compound with a more selective effect on rafts containing Aβ. Here, we showed that inhibitors of cPLA_2_, an enzyme that is implicated in tubule formation and intracellular trafficking [41-44], also affected Aβ_42_ distribution. The activation of cPLA_2_ is the first step in the production of PAF [61,62]. PAF is produced in neurons incubated with Aβ_42_, and the amounts of PAF were elevated in both the temporal cortex of patients with AD and in murine AD models [63]. Our study suggested that the effects of cPLA_2_ inhibitors on Aβ_42_ distribution were mediated by a reduction in PAF production; not only did PAF antagonists have similar effects as cPLA_2_ inhibitors, but PAF also reversed the effects of cPLA_2_ inhibitors on Aβ_42_ distribution and degradation.

Treating neurons with PLA_2_ inhibitors, PAF antagonists, or PAF did not affect total cellular cholesterol concentrations, suggesting that these drugs did not affect cholesterol synthesis. PAF receptors were concentrated in the ER, and PAF increased cholesterol concentrations within ER fractions. Our studies are consistent with the hypothesis that local cholesterol concentrations within the ER affect the formation and functional rafts involved in the trafficking of Aβ_42_. Consequently, PAF antagonists had similar effects to squalestatin on the trafficking of Aβ_42_ in that they reduced the amount of Aβ_42_ within rafts. PAF is involved in endocytosis and endosome formation [64,65], and the blockade of PAF receptors may block the formation of endocytic structures involved in the trafficking or Aβ_42_. In neurons pre-treated with PAF receptor antagonists, more Aβ_42_ was detected within lysosomes, sites of protein degradation [66], and the half-life of Aβ_42_ was reduced to less than 24 hours.

Notably, the PAF-induced increase in cholesterol was accompanied by reduced cholesterol esters, suggesting that cholesterol was being released from stores of cholesterol esters. The enzymes involved in the cholesterol ester cycle, ACAT and CEH, were also concentrated in the ER [67], and the PAF-induced increase in cholesterol was blocked by inhibition of CEH. The role of CEH in Aβ_42_ metabolism was demonstrated by using an inhibitor of CEH [48], which greatly increased the degradation of Aβ_42_ in a similar manner as PAF antagonists or squalestatin. Collectively these results indicate that PAF regulates the deacylation of cholesterol esters in the ER, freeing cholesterol that affects the formation and function of rafts which are involved in the trafficking of Aβ_42._

## Conclusions

**Figure 7:**
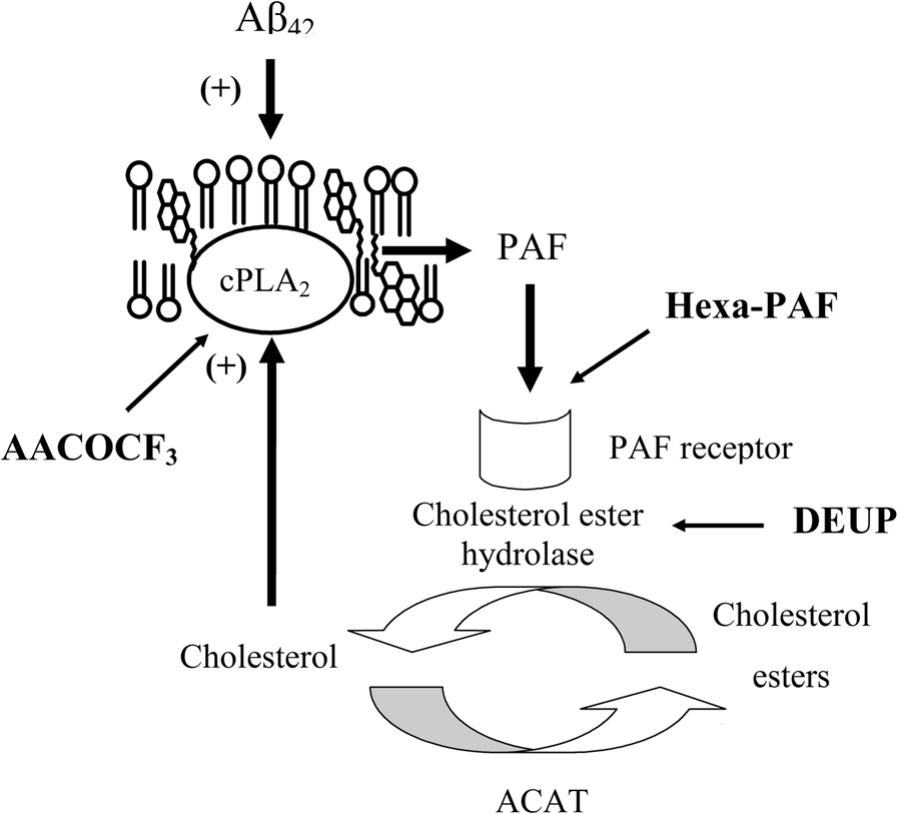
**Platelet-activating factor (PAF)-mediated hydrolysis of cholesterol esters stabilizes amyloid-beta (Aβ)-containing lipid rafts.** Schematic demonstrating the putative mechanism regulating the formation of Aβ-containing rafts. In this theory, Aβ_42_ activates cPLA_2_, resulting in the production of PAF. PAF activates cholesterol ester hydrolase (CEH) and the release of cholesterol from stores of cholesterol esters, which stabilizes Aβ_42_ in rafts. Inhibition of cPLA_2_ (arachidonyl trifluoromethyl ketone, or AACOCF_3_), antagonism of the PAF receptors (Hexa-PAF), or inhibition of CEH (diethylumbelliferyl phosphate, is DEUP) reduces the cholesterol concentrations in the endoplasmic reticulum (ER). cPLA_2_, cPLA2 is cytoplasmic phospholipase A_2_; Hexa-PAF, 1-O-hexadecyl-2-acetyl-*sn*-glycerol-3-phospho-(N,N,N-trimethyl)-hexanolamine.

In summary, these results support the hypothesis that Aβ_42_-induced activation of PLA_2_ leads to the production of PAF which releases cholesterol from cholesterol esters. The cholesterol helps stabilize Aβ_42_ in rafts which traffic via a recycling pathway that avoids the lysosomes. Thus, PAF receptor antagonists that reduce the release of cholesterol affect the rafts that direct Aβ_42_ into the recycling pathway as summarized in Figure 7. Consequently, in these neurons, the half-life of Aβ_42_ was greatly reduced. In neurons treated with cholesterol synthesis inhibitors, PAF antagonists, or CEH inhibitors, the trafficking of Aβ_42_ is altered so that Aβ_42_ was found outside rafts, was targeted to lysosomes, and rapidly degraded. The novel observation that the activation of specific signaling pathways controls the trafficking and degradation of Aβ_42_ may aid the development of drugs to delay the progression of AD.

## Abbreviations

7PA2-CM: conditioned media from 7PA2 cells; AACOCF3: arachidonyl trifluoromethyl ketone; ACAT: acyl-coenzyme A:cholesterol acyltransferase; AD: Alzheimer’s diesease; Aβ: amyloid-beta; CEH: cholesterol ester hydrolase; CM: conditioned media; cPLA2: cytoplasmic phospholipase A_2_; DEUP: diethylumbelliferyl phosphate; DRM: detergent-resistant membrane; ELISA: enzyme-linked immunosorbent assay; ER: endoplasmic reticulum; Hexa-PAF: 1-O-hexadecyl-2-acetyl-*sn*-glycerol-3-phospho-(N,N,N-trimethyl)-hexanolamine; LAMP-1: lysosome-associated membrane protein 1; mAb: monoclonal antibody; MAFP: methyl arachidonyl fluorophosphonate; PAF: platelet-activating factor; PBS: phosphate-buffered saline; PLA2: phospholipase A_2_; PVDF: polyvinylidiene fluoride; SDS: sodium dodecyl sulphate.

## Competing interests

The authors declare that they have no competing interests.

## Authors’ contributions

VI and CS contributed to data collection and analysis and to drafting and critical revision of manuscript. CB contributed to conception and design, data collection and analysis, and manuscript writing and revision. AW contributed to conception and design and to manuscript writing. All authors read and approved the final manuscript.
